# Genomic Epidemiology of Carbapenemase-Producing and Colistin-Resistant *Enterobacteriaceae* among Sepsis Patients in Ethiopia: a Whole-Genome Analysis

**DOI:** 10.1128/aac.00534-22

**Published:** 2022-07-25

**Authors:** Melese Hailu Legese, Daniel Asrat, Adane Mihret, Badrul Hasan, Amaha Mekasha, Abraham Aseffa, Göte Swedberg

**Affiliations:** a Department of Medical Laboratory Sciences, College of Health Sciences, Addis Ababa University, Addis Ababa, Ethiopia; b Armauer Hansen Research Institute, Addis Ababa, Ethiopia; c Department of Medical Biochemistry and Microbiology, Biomedical Centre, Uppsala University, Uppsala, Sweden; d Department of Microbiology, Immunology and Parasitology, College of Health Sciences, Addis Ababa University, Addis Ababa, Ethiopia; e Department of Pediatrics and Child Health, College of Health Sciences, Addis Ababa University, Addis Ababa, Ethiopia

**Keywords:** whole-genome sequencing, genomic epidemiology, carbapenemase producing, colistin resistance, sequence typing, phylogeny structure, plasmid replicons, *Enterobacteriaceae*, sepsis, Ethiopia

## Abstract

Sepsis due to carbapenemase-producing and colistin-resistant *Enterobacteriaceae* is a global health threat. A multicenter study was conducted between October 2019 and September 2020 at four hospitals located in different parts of Ethiopia. From a total of 1,416 sepsis patients, blood culture was performed. *Enterobacteriaceae* were confirmed using matrix-assisted laser desorption ionization–time of flight mass spectrometry (MALDI-TOF MS). Carbapenem and colistin susceptibility testing was performed using disk diffusion, broth microdilution, and Etest strip. *Enterobacteriaceae* isolates (*n* = 301) were subjected to whole-genome sequencing using Illumina HiSeq 2500. SPAdes version 3.9 was used for genome assembly. Carbapenem and colistin resistance genes, chromosomal point mutations, sequence types, and plasmid replicons were identified using tools at the Center for Genomic Epidemiology. Phylogeny structure was constructed using CSI Phylogeny 1.4. Visualization of trees and metadata was done using iTOL v6.5.2. Among 301 *Enterobacteriaceae*, 22 Klebsiella pneumoniae, 2 Klebsiella variicola, and 3 Enterobacter cloacae isolates showed reduced susceptibility to meropenem (7% of tested isolates). *bla*_NDM-1_, *bla*_NDM-5_, and *bla*_OXA-181_ were variants of carbapenemase genes detected. Co-occurrence of *bla*_NDM-5_ and *bla*_OXA-181_ was detected with 4 K. pneumoniae strains. K. pneumoniae and K. variicola showed chromosomal alterations of ompK36 and ompk37. Plasmid incompatibility (Inc) groups Col, IncC, IncHI, IncF, IncFII, IncR, and IncX3 were identified among carbapenem-resistant K. pneumoniae and E. cloacae isolates. Two *mcr-9* genes were detected from Salmonella species and K. pneumoniae. The dissemination of carbapenemase-producing *Enterobacteriaceae* in all hospitals is worrying. Multiple carbapenemase genes were detected, with *bla*_NDM_ variants the most frequent. The occurrence of colistin-resistant *Enterobacteriaceae* among sepsis patients is critical. Implementation of effective antimicrobial stewardship is urgently needed.

## INTRODUCTION

Sepsis is a severe public health problem with high rates of mortality in the absence of early recognition of the syndrome and early onset of appropriate antimicrobial therapy (1). It is a life-threatening condition resulting from a dysregulated immune response to the infection, which ultimately results in organ dysfunction (2, 3). Antibiotic options for the management of septic patients caused by carbapenemase-producing *Enterobacteriaceae* (CPE) are very narrow, which can lead to longer hospital stays, increased hospital costs, and increased mortality (1, 4). The spread of CRE is a current global public health threat (5, 6). Carbapenems are the last-line antibiotics for the treatment of infections caused by extended-spectrum β-lactamase-producing *Enterobacteriaceae* (7) and other multidrug-resistant *Enterobacteriaceae* (8).

In *Enterobacteriaceae*, enzyme production, efflux pumps, and porin mutations are major mechanisms of carbapenem resistance (7, 9). Carbapenem resistance in *Enterobacteriaceae* is primarily due to production of carbapenemase enzymes (10), which are categorized into Ambler classes A (serine penicillinases), B (metallo-β-lactamases), and D (oxacillinases) (11). Variants of K. pneumoniae carbapenemase (KPC) (12, 13), New Delhi metallo-β-lactamase (NDM) (14, 15), and carbapenem-hydrolyzing oxacillinase (OXA) (6, 16) are main carbapenemase enzymes (6).

*Enterobacteriaceae* that are resistant to carbapenems are treated with colistin, which is a last-resort drug used to treat infections caused by multidrug-resistant Gram-negative bacteria (17). However, the emergence and dissemination of plasmid-mediated mobile colistin resistance (*mcr*) genes are worsening the problem (17, 18). Colistin resistance in *Enterobacteriaceae* can be due to structural modifications of the bacterial lipopolysaccharide, chromosomal mutations, or plasmid-borne mobile colistin resistance genes (*mcr-1* to *mcr-10*) (7).

The World Health Organization (WHO) has listed carbapenemase-producing and colistin-resistant *Enterobacteriaceae* as critical priority pathogens (19, 20). These strains are spreading throughout the world and posing serious public health threats (21–24). However, there is a scarcity of data related to the genomic epidemiology of carbapenemase-producing and colistin-resistant *Enterobacteriaceae* in sub-Saharan countries, including Ethiopia. It is a crucial time to determine the genomic epidemiology of carbapenemase-producing and colistin-resistant *Enterobacteriaceae* at a larger scale in order to guide future antimicrobial resistance control programs. Hence, this study aimed to determine the molecular epidemiology of carbapenemase-producing and colistin-resistant *Enterobacteriaceae* among patients investigated for sepsis at four Ethiopian teaching/referral hospitals which are located in the central, southern, and northern parts of the country. These hospitals are serving millions of people in the surrounding catchment area and people who are referred to these hospitals.

## RESULTS

### Sociodemographic characteristics.

In the present study, a total of 1,416 patients investigated for sepsis from four different hospitals were enrolled. The number of patients from Tikur Anbessa Specialized Hospital (TASH) was 501, and the numbers from Yekatit 12 Specialized Hospital Medical College (Y12HMC), Dessie Referral Hospital (DRH), and Hawassa University Comprehensive Specialized Hospital (HUCSH) were 298, 301, and 316, respectively. Male participants made up 55.3%, while 46.7% were female. Patients’ ages ranged from half a day to 90 years with a mean age of 8.85 years (see Table S1 in the supplemental material).

### *Enterobacteriaceae* strains subjected to whole-genome sequencing.

Among 1,416 patients, a total of 301 *Enterobacteriaceae* strains were isolated from all study sites, and all these *Enterobacteriaceae* were subjected to whole-genome sequencing (WGS). Klebsiella pneumoniae (*n* = 103), Klebsiella variicola (*n* = 74), and Escherichia coli (*n* = 53) were most frequently identified, and their frequency varied between the four hospitals (Table 1).

**TABLE 1:** Frequency and distribution of *Enterobacteriaceae* species isolated from patients investigated for sepsis and subjected to whole-genome sequence in four Ethiopian hospitals^*a*^

*Enterobacteriaceae* species	No. (%) of isolates found at:			
	DRH	TASH	HUCSH	Y12HMC
Klebsiella pneumoniae (*n* = 103)	12 (11)	39 (42)	22 (39)	30 (75)
Klebsiella variicola (*n* = 74)	44 (39)	2 (2)	28 (50)	
Escherichia coli (*n* = 53)	17 (15)	28 (30)	4 (7)	4 (10)
Enterobacter cloacae (*n* = 21)	10 (9)	6 (7)	1 (2)	4 (10)
Pantoea dispersa (*n* = 21)	20 (18)	1 (1)		
Klebsiella oxytoca (*n* = 13)	5 (4)	6 (7)	1 (2)	1 (3)
Enterobacter xiangfangensis (*n* = 3)	3 (3)			
Raoultella ornithinolytica (*n* = 2)	1 (1)	1 (1)		
Serratia marcescens (*n* = 2)		2 (2)		
Leclercia adecarboxylata (*n* = 2)		2 (2)		
Enterobacter bugandensis (*n* = 1)		1 (1)		
Enterobacter kobei (*n* = 1)		1 (1)		
Enterobacter ludwigii (*n* = 1)		1 (1)		
Kosakonia cowanii (*n* = 1)	1 (1)			
Lelliottia amnigena (*n* = 1)		1 (1)		
Salmonella spp. (*n* = 1)				1 (3)
Shigella dysenteriae (*n* = 1)		1 (1)		
Total (*n* = 301)	113 (100)	92 (100)	56 (100)	40 (100)

a TASH, Tikur Anbessa Specialized Hospital; Y12HMC, Yekatit 12 Specialized Hospital Medical College; DRH, Dessie Referral Hospital; HUCSH, Hawassa University Comprehensive Specialized Hospital.

### Disk diffusion and MIC tests against carbapenems.

Among *Enterobacteriaceae* isolates (*n* = 301) initially tested against meropenem, 9% (*n* = 27/301) showed either resistant or intermediate levels of susceptibility. These *Enterobacteriaceae* isolates were repeatedly tested for meropenem resistance and simultaneously tested against imipenem and ertapenem by using disk diffusion. To investigate the ability of each test of the detection of carbapenem-resistant *Enterobacteriaceae*, all were further tested against meropenem by using broth microdilution and against meropenem, imipenem, and ertapenem by using Etest strips. Among *Enterobacteriaceae* isolates (*n* = 27) tested against imipenem, meropenem, or ertapenem by using disk diffusion, 21 showed reduced susceptibility to one or all of them. On the other hand, 23 of them showed reduced susceptibility against meropenem by use of microbroth MIC test, while, comparatively, the same number of *Enterobacteriaceae* isolates showed reduced susceptibility by using imipenem (*n* = 20) and meropenem (*n* = 21) Etest strip; 24 *Enterobacteriaceae* isolates showed reduced susceptibility for ertapenem Etest strip (Table 2).

**TABLE 2:** *Enterobacteriaceae* isolates that showed reduced susceptibility against carbapenems using disk diffusion, broth microdilution, and Etest strip^*a*^

Isolate no.	*Enterobacteriaceae* species	Results of:													
		Disk diffusion						Broth microdilution		Etest strip					
		ERT susceptibility	ERT ZD (mm)	IMP susceptibility	IMP ZD (mm)	MEP susceptibility	MEP ZD (mm)	Susceptibility	MIC level (μg/mL)	ERT susceptibility	ERT-MIC (μg/mL)	IMP susceptibility	IMP MIC (μg/mL)	MEP susceptibility	MEP MIC (μg/mL)
1	K. pneumoniae	I	20	R	14	R	14	I	2	R	3	R	4	R	4
2	K. pneumoniae	S	25	R	15	R	15	I	2	R	>32	S	<0.125	S	1
3	K. pneumoniae	I	19	R	16	R	16	R	16	R	>32	I	3	R	4
4	K. pneumoniae	S	30	S	25	S	26	R	32	S	<0.125	S	<0.125	S	<0.125
5	K. pneumoniae	R	15	R	15	R	12	R	16	R	2	R	6	I	2
6	K. pneumoniae	R	10	I	20	R	15	R	32	R	>32	R	>32	R	>32
7	K. pneumoniae	R	6	R	16	R	8	R	32	R	>32	R	>32	R	>32
8	K. pneumoniae	R	16	I	20	R	18	R	16	R	4	S	<0.125	I	2
9	K. pneumoniae	I	19	I	20	I	21	R	8	R	>32	S	<0.125	I	2
10	K. pneumoniae	S	22	I	22	I	21	R	32	R	2	S	<0.125	S	1
11	K. pneumoniae	R	6	R	16	R	6	R	32	R	>32	R	>32	R	>32
12	K. pneumoniae	S	28	S	30	S	30	R	16	S	<0.125	S	<0.125	S	<0.125
13	K. pneumoniae	R	6	R	15	R	6	R	>32	R	>32	R	4	R	>32
14	K. pneumoniae	R	6	R	16	R	8	R	>32	R	>32	R	>32	R	>32
15	K. pneumoniae	R	6	R	15	R	6	R	>32	R	>32	R	>32	R	>32
16	K. pneumoniae	R	6	R	10	R	6	R	>32	R	>32	R	>32	R	>32
17	K. pneumoniae	R	6	I	20	R	15	R	16	R	16	R	16	R	8
18	K. pneumoniae	R	8	R	15	R	10	R	16	R	>32	R	>32	R	>32
19	K. pneumoniae	S	30	S	30	S	31	S	<0.003	S	0.25	I	2	S	0.5
20	K. pneumoniae	R	6	R	15	R	10	R	>32	R	>32	R	>32	R	>32
21	K. pneumoniae	S	27	S	31	S	30	S	1	R	>32	R	>32	R	8
22	K. pneumoniae	R	15	S	26	S	24	R	>32	R	>32	R	>32	R	>32
23	K. variicola	S	33	S	30	S	30	S	0.25	I	1	S	0.19	S	0.125
24	K. variicola	S	30	S	30	S	30	S	0.065	R	>32	R	>32	R	>32
25	E. cloacae	R	6	R	18	R	10	R	32	R	>32	R	>32	R	>32
26	E. cloacae	R	8	R	19	R	13	R	16	R	2	R	8	R	6
27	E. cloacae	R	6	R	19	R	12	R	16	R	>32	R	>32	R	>32
Total		19		20		20		23		24		20		21	

a S, sensitive; I, intermediate; R, resistant; ZD, zone diameter; ERT, ertapenem; MEP, meropenem; IMP, imipenem.

Among all *Enterobacteriaceae*, only 22 K. pneumoniae, 2 K. variicola, and 3 E. cloacae isolates showed reduced susceptibility to meropenem from the initial testing. The disk diffusion showed that 18 K. pneumoniae and 3 E. cloacae isolates had reduced susceptibility to imipenem, meropenem, or ertapenem alone or all of them (Table 2). On the other hand, 20 K. pneumoniae and 3 E. cloacae isolates showed reduced susceptibility against meropenem using broth meropenem MIC test (Table 2). K. variicola (*n* = 2) showed reduced susceptibility to one of meropenem, imipenem, or ertapenem Etest strip only. One of the two K. variicola isolates initially scored as resistant to meropenem then showed full susceptibility according to the repeated disk test, but it showed an intermediate level according to Etest. The other K. variicola isolate also showed susceptibility to all carbapenems during the disk test but full resistance according to Etest (Table 2).

### Molecular epidemiology of carbapenemase-producing *Enterobacteriaceae*.

Including *Enterobacteriaceae* isolates that showed reduced susceptibility to any of the carbapenem drugs, all *Enterobacteriaceae* isolated from sepsis patients were processed using whole-genome sequencing. The highest proportion of carbapenemase-producing *Enterobacteriaceae* was detected at TASH (16%), which is located centrally in Ethiopia (Table 3). Many of the carbapenemase genes encoding *Enterobacteriaceae* were identified among male patients (9%), patients who had underlying diseases (15%), and patients who were referred from other health facilities to the study sites (11%). Of *Enterobacteriaceae* obtained from pediatric wards (*n* = 68), 15% had at least one carbapenemase gene (Table 4). Among patients who showed blood culture positivity for *Enterobacteriaceae*, possible risk factors for carbapenemase-producing *Enterobacteriaceae* were assessed. However, the adjusted odds ratio showed that none of these risk factors had a statistically significant association with the acquisition of carbapenemase-producing *Enterobacteriaceae*.

**TABLE 3:** Frequency and distribution of carbapenemase-coding genes detected at four Ethiopian hospitals^*a*^

Carbapenemase gene	Total no. (%) of genes detected	No. (%) of genes detected at:			
		DRH	HUCSH	TASH	Y12HMC
*bla*_NDM_ type	20 (7)	2 (2)	1 (2)	15 (16)	2 (5)
*bla*_NDM-1_	16 (5)^*b*^	2 (2)	1 (2)	11 (12)	2 (5)
*bla*_NDM-5_	4 (1)			4 (4)	
*bla*_OXA_ type	4 (1)			4 (4)	
*bla*_OXA-181_	4 (1)			4 (4)	

a TASH, Tikur Anbessa Specialized Hospital; Y12HMC, Yekatit 12 Specialized Hospital Medical College; DRH, Dessie Referral Hospital; HUCSH, Hawassa University Comprehensive Specialized Hospital.

b Detected frequently.

**TABLE 4:** Frequency of *Enterobacteriaceae* isolates that harbored at least one carbapenemase gene in relation to patient characteristics^*a*^

Patient characteristics	No. (%) of carbapenemase producers	No. (%) of carbapenemase nonproducers
Hospital		
DRH (*n* = 113)	2 (2)	111 (98)
TASH (*n* = 92)	15 (16)	77 (84)
HUCSH (*n* = 56)	1 (2)	55 (98)
Y12HMC (*n* = 40)	2 (5)	38 (95)
Gender		
Male (*n* = 174)	16 (9)	158 (91)
Female (*n* = 127)	4 (3)	123 (97)
Age category		
≤29 days (*n* = 187)	5 (3)	182 (97)
30 days to ≤1 yr (*n* = 32)	5 (16)	27 (84)
>1 to ≤5 yrs (*n* = 21)	4 (19)	17 (81)
>5 to <18 yrs (*n* = 22)	4 (18)	18 (82)
≥18 yrs (*n* = 39)	2 (5)	37 (95)
Ward		
EOPD (*n* = 12)	0 (0)	12 (100)
ICU (*n* = 8)	1 (13)	7 (87)
Medical ward (*n* = 15)	3 (20)	12 (80)
NICU (*n* = 189)	5 (3)	184 (97)
Pediatrics (*n* = 68)	10 (15)	58 (85)
Surgical ward (*n* = 9)	1 (11)	8 (89)
Hospital stay duration		
1 wk (*n* = 194)	5 (3)	189 (97)
2 wks (*n* = 37)	3 (8)	34 (92)
3 wks (*n* = 23)	3 (13)	20 (87)
≥4 wks (*n* = 47)	9 (19)	38 (81)
Underlying diseases		
Yes (*n* = 117)	15 (13)	102 (87)
No (*n* = 184)	6 (3)	178 (97)
Previous hospitalization		
Yes (48)	9 (19)	39 (81)
No (*n* = 253)	11 (4)	242 (96)
Referral patient^*b*^		
Yes (*n* = 140)	12 (9)	128 (91)
No (*n* = 161)	8 (5)	153 (95)
Previous antibiotic treatment history		
Yes (*n* = 52)	6 (12)	46 (88)
No (*n* = 249)	14 (6)	234 (94)

a TASH, Tikur Anbessa Specialized Hospital; Y12HMC, Yekatit 12 Specialized Hospital Medical College; DRH, Dessie Referral Hospital; HUCSH, Hawassa University Comprehensive Specialized Hospital; EOPD, emergency outpatient department.

b Patients who were transferred from other health care facilities to the study sites.

### Carbapenemase genes.

Variants of *bla*_NDM_ and *bla*_OXA_ were carbapenemase genes detected among *Enterobacteriaceae* sequenced from all study sites (Table 3). Of all *Enterobacteriaceae* subjected to WGS, 7% (*n* = 20/301) of strains carried at least one carbapenemase gene. Of the 20 *Enterobacteriaceae* strains (17 K. pneumoniae and 3 E. cloacae), 15 were identified at TASH, which is located in the central part of Ethiopia. Of the carbapenemase genes, *bla*_NDM_ (7%) was widely disseminated and detected in all hospitals. *bla*_NDM-1_ was the most frequent carbapenemase gene, with an overall detection rate of 5%. It was detected more frequently at TASH (13%), while its detection in the northern and southern hospitals was 2% each. All *bla*_NDM-5_ genes were detected at TASH and accounted for 4%, but they were not detected in the other three hospitals (Table 3). *bla*_OXA-181_ was the only variant of the *bla*_OXA_ carbapenemase gene detected at 1% overall frequency, and all were detected centrally at TASH. No *bla*_OXA_ carbapenemase gene variants were detected in the northern and southern parts of the country (Table 3).

**FIG 1:**
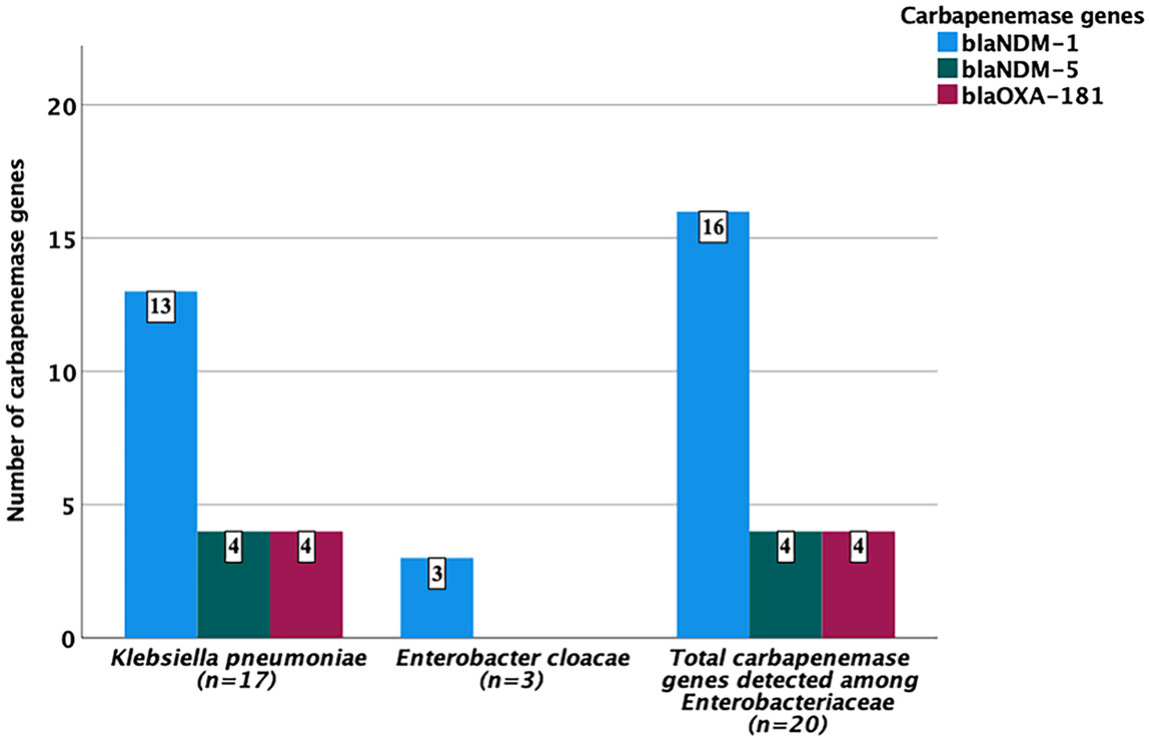
Frequency and distribution of carbapenemase genes against *Enterobacteriaceae*.

While *bla*_NDM-1_ was detected among both K. pneumoniae and E. cloacae, all *bla*_NDM-5_ genes were carried only by K. pneumoniae (Fig. 1). Co-occurrence of multiple carbapenemase genes was only detected with 4 K. pneumoniae that carried *bla*_NDM-5_ and *bla*_OXA-181_ concurrently. These 4 K. pneumoniae strains were identified only at TASH in its pediatric and neonatal intensive care unit (NICU) wards.

### Carbapenem resistance due to chromosomal point mutations.

**FIG 2:**
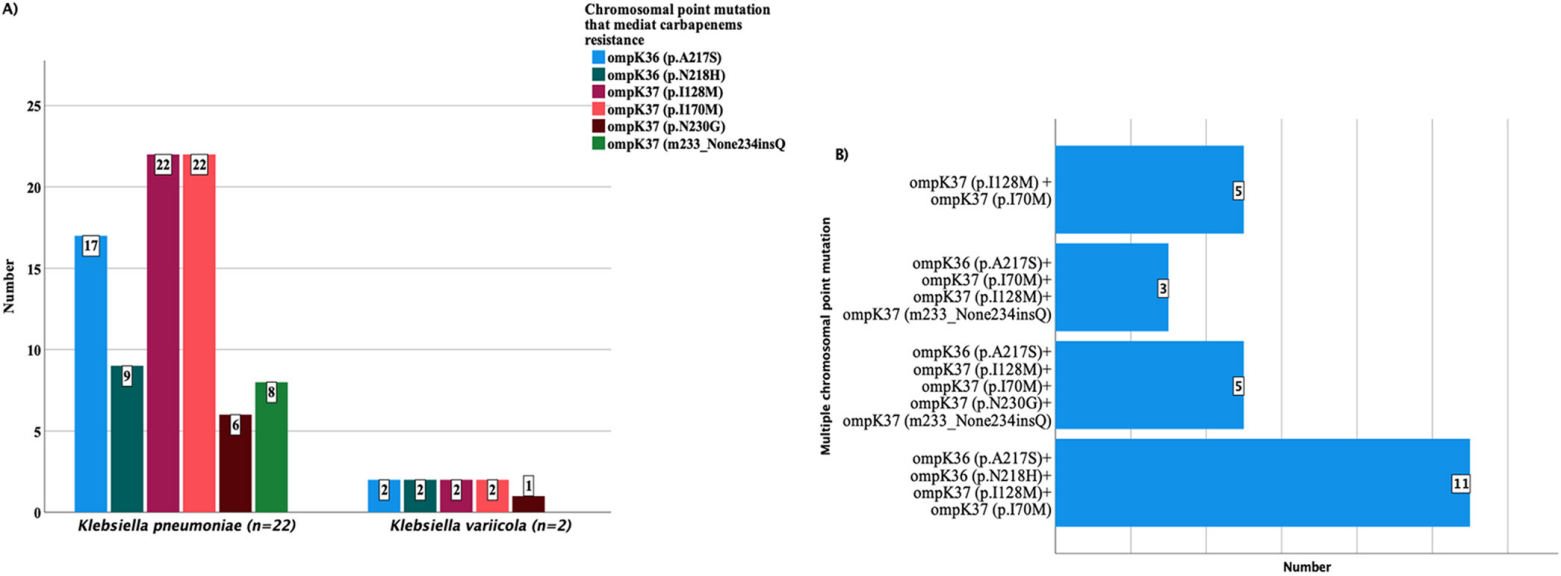
Frequency (A) and multiple occurrences (B) of chromosomal point mutations with K. pneumoniae and K. variicola that showed reduced susceptibility to carbapenems by the disk diffusion or MIC test.

Since some *Enterobacteriaceae* strains showed reduced susceptibility to carbapenems phenotypically but carried no carbapenemase genes, all strains were further assessed for the presence of chromosomal point mutations that could mediate carbapenem resistance. In addition to the acquired carbapenemase genes, all K. pneumoniae (*n* = 22) *and* K. variicola (*n* = 2) isolates that showed reduced susceptibility to carbapenems by disk diffusion or MIC tests were also assessed for the presence of chromosomal point mutations. All of these were found to have at least two chromosomal point mutations, ompK36 (p.A217S and p.N218H) and ompk37 (p.I128M, p.i170M, p.N230G, and m233_None234insQ) (Fig. 2A). Most point mutations appeared concurrently (Fig. 2B). It was not possible to screen E. cloacae for chromosomal point mutations since, currently, there is no point mutation database in the ResFinder designed for this strain. Other antimicrobial resistance genes detected among K. pneumoniae (*n* = 22), E. cloacae (*n* = 3), and K. variicola (*n* = 2) are shown in Table S2.

### Genetic diversity and population structure.

The sequence types (STs) of 22 K. pneumoniae and 3 E. cloacae strains that showed reduced susceptibility to carbapenems by using phenotypic methods were identified. K. pneumoniae strains were clustered into 11 different STs (Fig. S1). ST14 and ST437 of K. pneumoniae were recognized as high-risk clones, while ST101 and ST883 were epidemiologically important. E. cloacae (*n* = 3) isolates were clustered into ST182 and ST231 and isolated at TASH only in its pediatric and surgical wards. It was not possible to identify the sequence type of K. variicola since it is not included in the multilocus sequence typing (MLST) database currently.

**FIG 3:**
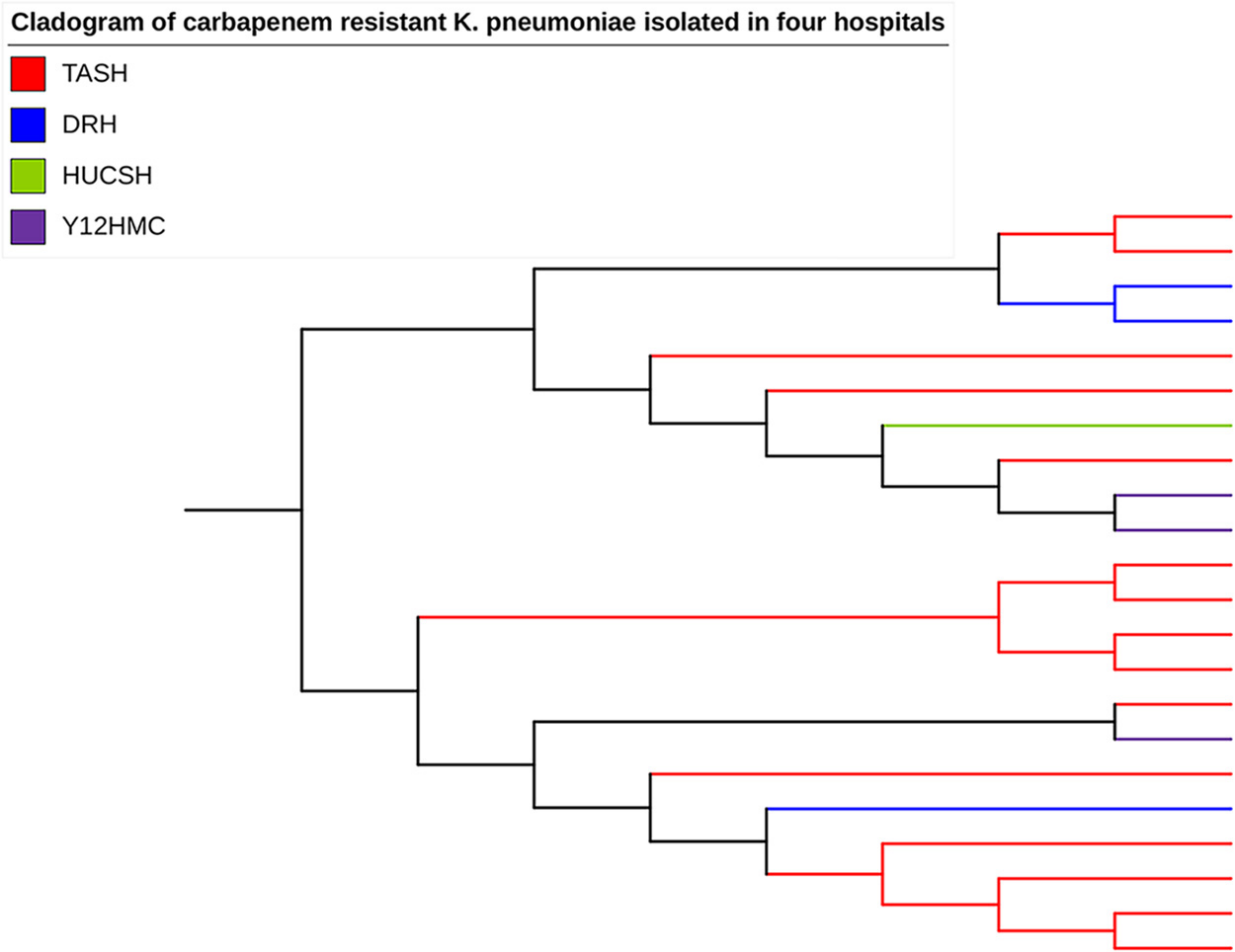
Phylogenetic tree generated from the dynamic core genome of 22 carbapenem-resistant K. pneumoniae isolates from four Ethiopian referral hospitals.

**FIG 4:**
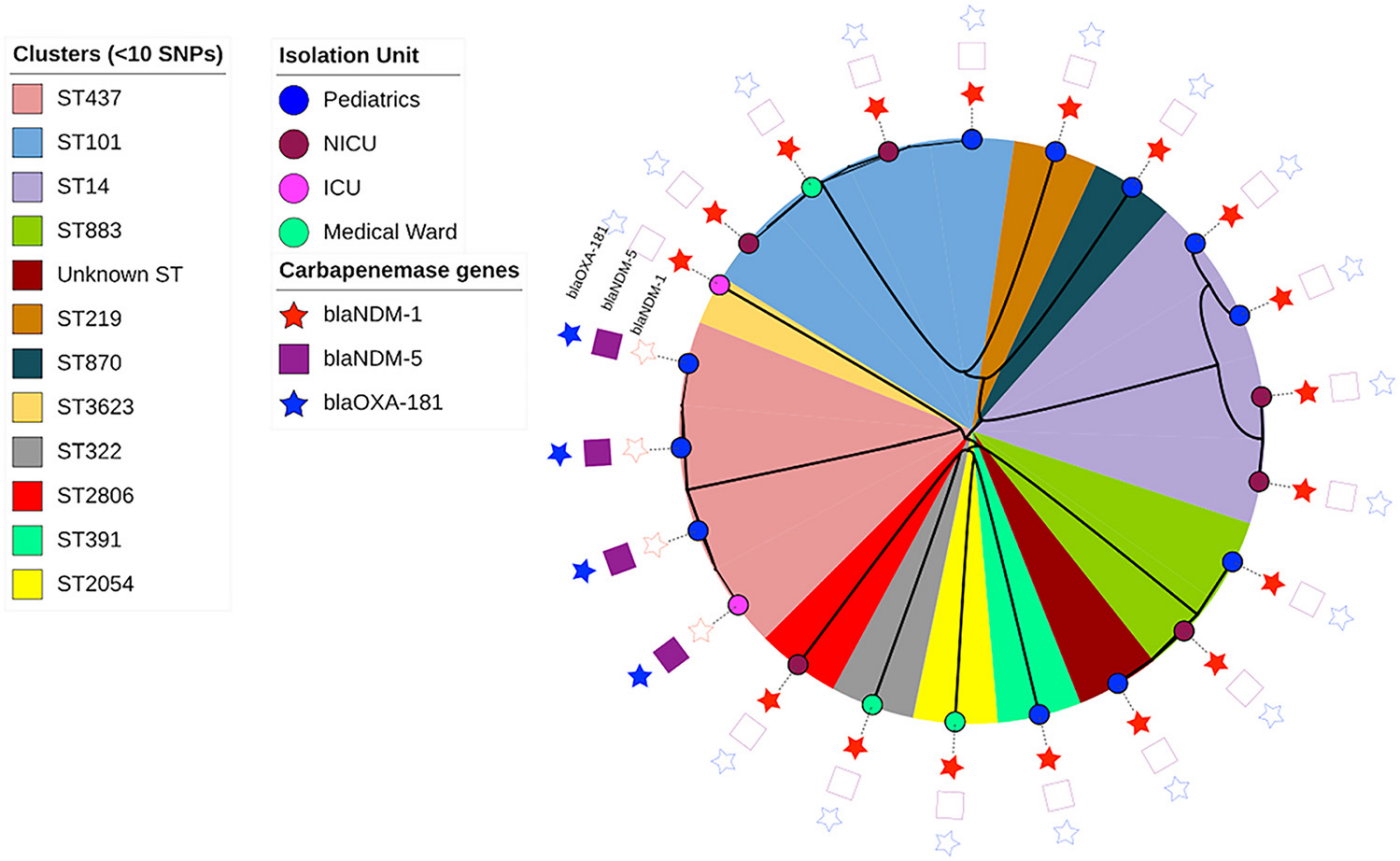
Clonal clusters of the diverse sequence types of carbapenem-resistant K. pneumoniae (*n* = 22) isolated from NICU, pediatric, medical, and ICU wards. SNP clusters showing the distribution of related and unrelated clones of carbapenemase gene-carrying K. pneumoniae isolates (*n* = 17) in different wards and of different sequence types.

**FIG 5:**
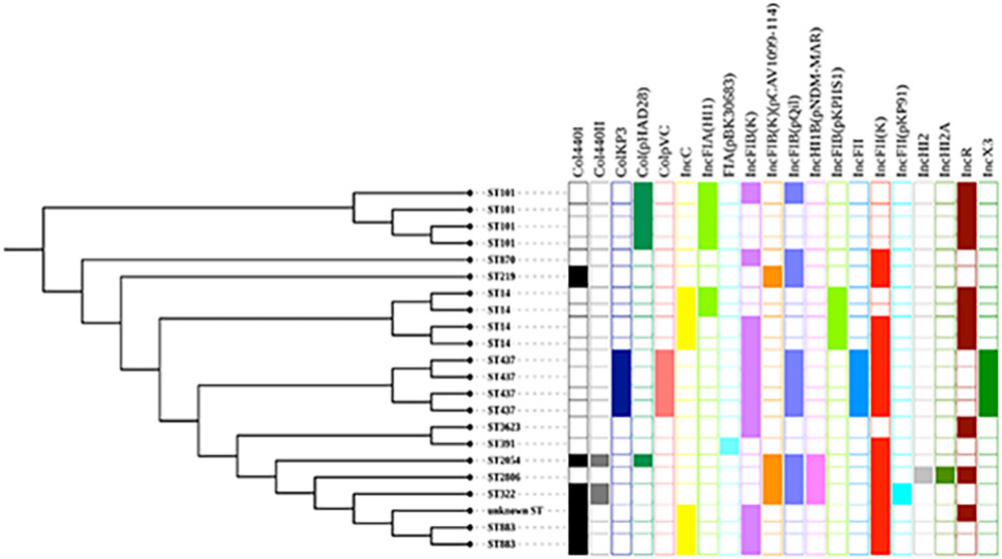
Plasmid contents of carbapenem-resistant K. pneumoniae isolates (*n* = 22).

The population structures of all carbapenem-resistant K. pneumoniae isolates (*n* = 22) were constructed using a maximum-likelihood tree inferred from their dynamic core genome (Fig. 3). Some clonally related strains were distributed in the same ward, while a few unrelated clones circulated in different wards (Fig. 4). *Bla*_NDM-1_ was encoded by both related and unrelated clones of K. pneumoniae that were isolated from different wards. *Bla*_NDM-5_- and *bla*_OXA-181_-carrying ST437 isolates of K. pneumoniae were related clones isolated in pediatric and ICU wards (Fig. 5).

### Plasmid content and linkage to carbapenemase genes.

All carbapenem-resistant and carbapenemase-producing K. pneumoniae (*n* = 22) carried multiple plasmid replicons (Fig. 5). Plasmid incompatibility (Inc) groups IncC, IncHI, IncF, IncR, and IncX3 were identified among carbapenem-resistant K. pneumoniae (Fig. 5). The plasmid IncF group was the most frequent type of plasmid replicon and included IncFIA(HI1), IncFIB(K), IncFIB(K) (pCAV1099-114), IncFIB(pQil), IncFIB(pKPHS1), IncFII, IncFII(K), and IncFII(pKP91) (Fig. 5). Plasmid group IncHI (IncHI2 and IncHI2A) was only carried by one, K. pneumoniae ST2806, while the other single case of plasmid occurrence was FIA(pBK30683) carried by K. pneumoniae ST391.

The WGS data with short reads made it difficult to link all carbapenemase genes to specific plasmids; however, some patterns could be identified. Six *bla*_NDM-1_ carriers gave long contigs (>90,000 bp), and the IncC determinant according to PlasmidFinder was located in the same contig as the resistance gene. The most similar plasmid in the GenBank was K. pneumoniae strain 1027_incC plasmid p1027_NDM_IncC (GenBank accession no. MZ606384). Three of the host strains belonged to ST14, and the others belonged to ST883 (Fig. 5). One of the ST883 strains differed somewhat from the rest and was scored as an “unknown” ST, most closely related to ST883 (Fig. 5). There was one more ST14 strain that carried an IncC plasmid, but the quality of the sequence result was lower than for the others, and the *bla*_NDM-1_ gene could not be unequivocally linked to the Inc determinant. However, the sequence similarities were good enough to make the IncC plasmid the most likely location for the *bla*_NDM-1_ gene. Another four samples belonging to ST101 gave contigs of intermediate length that could be linked up to a common contig of 26,486 bp, and all carried a plasmid belonging to IncR (Fig. 5). In these ST101 strains, the *bla*_NDM-1_ gene was located on the same contig as the IncR determinant. The remaining samples with shorter contigs were more difficult to determine, but in two more samples with short contigs lacking the IncR plasmid, linkage to an IncF plasmid determinant was found. These two samples were of different STs, 2054 and 332. All *bla*_NDM-1_ carriers had the common immediate surrounding to the *bla*_NDM-1_ gene as shown in Fig. S2. All four examples of *bla*_NDM-5_ carriage were closely related in DNA sequence, and all host strains belonged to ST437. The sequence surrounding the *bla*_NDM-5_ gene most closely aligned with E. coli M719 plasmid pM71901 and covered a large stretch with many other resistance genes in a typical integron structure (GenBank accession no. AP023434). The sequences from all these isolates also contained an IncFII determinant surrounded by sequence identity with the structure shown in GenBank accession no. AP023434.

All carbapenemase-producing E. cloacae isolates also carried several types of plasmid replicons. Two E. cloacae isolates carried a similar set of multiple plasmids, Col(pHAD28), IncFIA(HI1), IncFIB(pECLA), IncFII(Yp), and IncFII(pECLA). The third E. cloacae strain also carried multiple plasmids but of different types [pCol440I, IncFII(Yp), and IncFII(pMET)]. However, the sequence information available was not good enough to unambiguously assign the resistance gene carrying contigs to a particular plasmid, and at least in one case, the sequence data showed a chromosomal insertion of the resistance gene. The sequence surrounding the *bla*_NDM-1_ gene matched perfectly the E. cloacae strain 174, found in databases as GenBank accession no. CP020528. Even if the two other strains carried plasmids very similar to Enterobacter cloacae strain WCHECl-14653, plasmid pNDM1_EC14653 (GenBank accession no. KP868647), it was not possible to link the *bla*_NDM-1_ gene with the plasmid by sequence analysis. Similarly, the plasmid content of K. variicola was assessed even though no acquired carbapenemase genes were detected, and one of the strains carried a single plasmid type IncM1, while the other K. variicola strain did not carry any plasmid replicon.

### Molecular epidemiology of colistin-resistant *Enterobacteriaceae*.

Initially, all *Enterobacteriaceae* isolates (*n* = 27) that showed reduced susceptibility against meropenem (10 μg) were tested for colistin susceptibility using the broth MIC test. All *Enterobacteriaceae* (*n* = 27) tested against colistin showed sensitivity with MIC levels of 0.25 μg/mL and below. On the other hand, among all whole-genome-sequenced *Enterobacteriaceae* strains (*n* = 301), detection of colistin resistance genes was only 0.7%, and this represents *mcr-9* that was detected in two hospitals located in the central and northern parts of the country. One case was from a Salmonella species (unclassified based on WGS) that was detected at Y12HMC, which is in central Ethiopia. The other *mcr-9* gene from a K. pneumoniae isolate was detected in the northern part at DRH. These two strains were tested against colistin phenotypically using the broth MIC test; however, both showed sensitivity to colistin. Both were also scored as carbapenem susceptible. These *mcr-9*-carrying strains showed no carbapenemase genes, while only K. pneumoniae showed a chromosomal point mutation of ompK37 (p.I128M) and ompK37 (p.I70M) that could mediate resistance to carbapenems. The K. pneumoniae that harbored *mcr-9* was of ST2806 and carried multiple plasmids, IncFIB(K) (pCAV1099-114), IncFIB(pNDM-Mar), IncFIB(pQil), IncFII(K), IncHI2, IncHI2A, and IncR. The sequence typing of the *mcr-9*-carrying Salmonella species was matched with ST534. This Salmonella species carried multiple plasmid replicon types of Col(pHAD28), IncHI2A, IncQ1, and IncY.

## DISCUSSION

The spread of carbapenemase-producing *Enterobacteriaceae* (CPE) is a current global public health threat. The burden of CPE among sepsis patients is considerably more severe in developing countries. Hence, this study attempted to describe the genomic epidemiology of CPE among sepsis patients in teaching/referral hospitals located in central, southern, and northern parts of Ethiopia. Among whole-genome-sequenced *Enterobacteriaceae* (*n* = 301), the overall genomic detection of CPE that carried at least one carbapenemase gene was 7% (*n* = 20). Our finding was in line with a study from India that reported 6% CPE (25). However, a higher occurrence of CPE was reported in Egypt (26). Several studies across the globe showed the spread of CPE in hospitals (6, 25–28). Similarly, our findings showed the spread of CPE with different magnitudes in Ethiopian referral hospitals. The burden of CPE in developing countries could be very serious due to limitation in resources and infrastructures to identify and implement necessary measures to control the spread of CPE.

Of the four hospitals included in this study, 15 of the 20 CPE species were identified at TASH, which is located centrally in Ethiopia. Of the 15 CPE species, 4 carried multiple carbapenemase genes. This hospital is the largest specialized hospital in the country and gives tertiary care to patients referred from all over the country. Since it is the main destination of patients referred from all parts of the country, who could carry CPE, to the hospital, this could be the main reason for the spread of CPE at this hospital. Moreover, the higher prescription rate of antibiotics, including meropenem, mostly influenced by the excessive number of patients referred to the hospital, could be a factor in the occurrence of CPE in the hospital (29, 30). Prior carbapenem use leading to the acquisition of *bla*_NDM-1_ strains (22) that ultimately increased the mortality rate of sepsis patients was documented (1). The finding of several identical or very closely related isolates at TASH could be an indication that the CPE are circulating in the hospital. For instance, all four isolates with *bla*_NDM-5_ were found in TASH, and they were identical. Likewise, all three ST883 isolates were found here. This finding specifically calls for strict antimicrobial stewardship at the hospital to control further spread of CPE inside the hospital and from the hospital to the community. At Y12HMC, the other centrally located hospital, detection of CPE was low (5% of the hospital isolates). The difference in CPE detection rate between Y12HMC and TASH might be due to variation in the number of referral cases and prescription rate, which are high at TASH. As stated above, referral cases and prescription rates of antibiotics could influence the occurrence and spread of CPE in a hospital setting. The variation of CPE circulation between hospitals showed the need for site-specific antimicrobial stewardship, even if they are located in the same area. CPE were also identified in other hospitals from the southern and northern parts of Ethiopia; however, their genomic frequency was low (2% each). The dissemination of CPE in all Ethiopian referral hospitals is worrying, while the lower genomic detection of CPE in the southern and northern hospitals has public health relevance.

Among all *Enterobacteriaceae* isolates sequenced, variants of *bla*_NDM_ and *bla*_OXA_ were the carbapenemase gene families detected in this study. The *bla*_NDM_ type (7%) was detected in all hospitals but was widely disseminated at TASH, with *bla*_NDM_-1 and *bla*_NDM-5_ as the two identified variants. Overall, *bla*_NDM-1_ (5%) was the most frequent carbapenemase gene detected that showed similarity with other studies across the globe (8, 30–34). A majority of *bla*_NDM-1_ was detected at TASH, where it amounted to 13% of all isolates, while its detection in the northern and southern regions was rare (2% each). The dissemination of *bla*_NDM_ was similar to findings from other African countries (8, 30, 32, 33, 35–37); however, a lower detection of *bla*_NDM_ was reported in the United States (38). All cases of *bla*_NDM-5_ and *bla*_OXA-181_ genes were detected at TASH and were always carried concomitantly, which showed similarity to a study from India (26). The occurrence of *bla*_OXA-48_-like carbapenemase genes also showed similarity to studies from other African countries (8, 32, 39) and India (26). While the *bla*_KPC_ type of carbapenemase gene was reported in Egypt (35), China (6, 32), the United States (36), and Portugal (37), no *bla*_KPC_ carbapenemase genes were detected in this study, which has public health significance.

In the current study, chromosomal alteration/mutations that could mediate carbapenem resistance were detected at ompK36 and ompk37. These chromosomal alterations were detected from all strains that showed reduced susceptibility to carbapenems using disk diffusion or microdilution and Etest strip MIC tests. Most of these chromosomal alterations occurred concurrently, similar to a finding from Nigeria (7). The occurrence of multiple chromosomal alterations could contribute a significant role in resistance to carbapenems and have clinical significance, even if the transferability to other strains is not as serious as with plasmid-mediated carbapenemase genes (7).

Among all *Enterobacteriaceae* species sequenced, K. pneumoniae (*n* = 17) and E. cloacae (*n* = 3) were found carrying carbapenemase genes, but none of the other *Enterobacteriaceae* isolates were. Among K. pneumoniae isolates, 13 carried *bla*_NDM-1_, and 4 carried *bla*_NDM-5_ and *bla*_OXA-181_ concurrently. Several studies across the globe (6, 27, 33, 37) reported that K. pneumoniae is a key carrier of carbapenemase genes. Including those isolates that were carbapenem resistant but devoid of carbapenemase genes, MLST analysis of all K. pneumoniae strains (*n* = 22) showed 11 sequence types (STs) where one was a novel ST. ST14 and ST437 (4 of each) were high-risk clones of K. pneumoniae identified, where all ST14s carried *bla*_NDM-1_, and all ST437s carried all cases of *bla*_NDM-5_ and *bla*_OXA-181_ concurrently. The occurrence of *bla*_NDM-1_-carrying ST14 and the coexistence of *bla*_NDM-5_ and *bla*_OXA-181_ among septicemic patients had similarity to a study from India (27). Identification of the high-risk clones of ST437 was documented in a study from China (38). The phylogenetic analysis of carbapenemase-carrying K. pneumoniae (*n* = 17) showed that some of these clones were related, while some others were unrelated clusters. The presence of *bla*_NDM-1_, *bla*_NDM-5_, and *bla*_OXA-181_ in high-risk international clones underlines the spread of CPE across the globe.

In our study, the other CPE identified was E. cloacae (*n* = 3), and all were found at TASH and carried *bla*_NDM-1_. Two E. cloacae isolates were typed as ST182 and were isolated in the pediatric ward, while the other identified as ST231 was isolated in the surgical ward. The occurrence of *bla*_NDM-1_ carried by an ST182 E. cloacae in clinical settings was similar to a study from Mexico (39); however, a different ST E. cloacae was reported from China (34).

Among *Enterobacteriaceae* that showed reduced susceptibility to one of the carbapenem drugs, multiple types of plasmids were detected from 22 K. pneumoniae and 3 E. cloacae isolates, while only one K. variicola isolate carried a single plasmid replicon. Plasmid incompatibility (Inc) groups IncC, IncHI, IncF, IncR, and IncX3 were identified among carbapenem-resistant K. pneumoniae isolates, where the plasmid IncF group was the most frequent type and included IncFIA(HI1), IncFIB(K), IncFIB(K) (pCAV1099-114), IncFIB(pQil), IncFIB(pKPHS1), IncFII, IncFII(K), and IncFII(pKP91). Several studies showed similar findings (8, 14, 40, 41). Even if all strains described here carry multiple plasmids, not all *bla*_NDM-1_ genes could be unambiguously assigned to a particular plasmid. The best associations possible were with one IncC and one IncR plasmid, but in a few isolates, linkage to an IncF determinant was found. Likewise, for all isolates carrying the *bla*_NDM-5_ determinant, there was linkage to an IncF determinant. For one E. cloacae strain, the most likely interpretation of the sequence data was a chromosomal insertion of the resistance gene. In all cases, the resistance genes were found in similar clusters surrounded by repeated elements, suggestive of a transposon environment. With our short-read approach, it was difficult to link the contig carrying the resistance gene with contigs assigned to the plasmid. More definite results could be obtained by transfer experiments or by long-read plasmid sequencing, both of which are planned for a more in-depth study of the plasmids carried by these strains.

In this study, the whole-genome analysis showed the occurrence of two cases of the colistin resistance genes *mcr-9* at a rate of 0.7%, detected in two hospitals located in the central (Y12HMC) and northern (DRH) parts of the country. The two *mcr-9*-encoded strains were one Salmonella species and one K. pneumoniae, both of which were reported as *mcr* carriers in different studies (42, 43). Though the detection rate was minimal, this first report showed the emergence of colistin resistance genes among sepsis patients in Ethiopian referral hospitals. Following *mcr-1*, *mcr-9* was reported as one of the most widely distributed colistin resistance genes in the globe with more than half of the cases detected in the United States (44). A conjugation experiment performed in China proved the successful transferability of *mcr-9* to other *Enterobacteriaceae* (45). In this study, all CRE and *mcr*-9 carrying strains showed susceptibility against colistin when tested using standard broth microdilution. This was a similar finding to a study from China that reported two *mcr-9*-harboring Enterobacter strains, which were susceptible to colistin (34). This further supports that *mcr-9* only causes colistin resistance under induction of expression (34). The detection of colistin-resistant *Enterobacteriaceae* among sepsis patients in Ethiopian referral hospitals is a public health threat. On the other hand, that no *mcr-1* to *mcr-4* genes were detected has public health relevance and supports the use of colistin for treatment.

### Conclusion.

The dissemination of carbapenemase-producing *Enterobacteriaceae* in the four hospitals is worrying. Multiple carbapenemase genes were detected, with *bla*_NDM_ variants as the most frequent. High-risk clones of K. pneumoniae that carried carbapenemase genes were identified frequently. The occurrence of colistin-resistant *Enterobacteriaceae* among sepsis patients is very serious. Multiple types of plasmid replicons were identified among *Enterobacteriaceae* isolates carrying carbapenemase and colistin resistance genes. The current findings strongly suggest the urgent need for effective antimicrobial stewardship.

## MATERIALS AND METHODS

### Study design and study sites.

**FIG 6:**
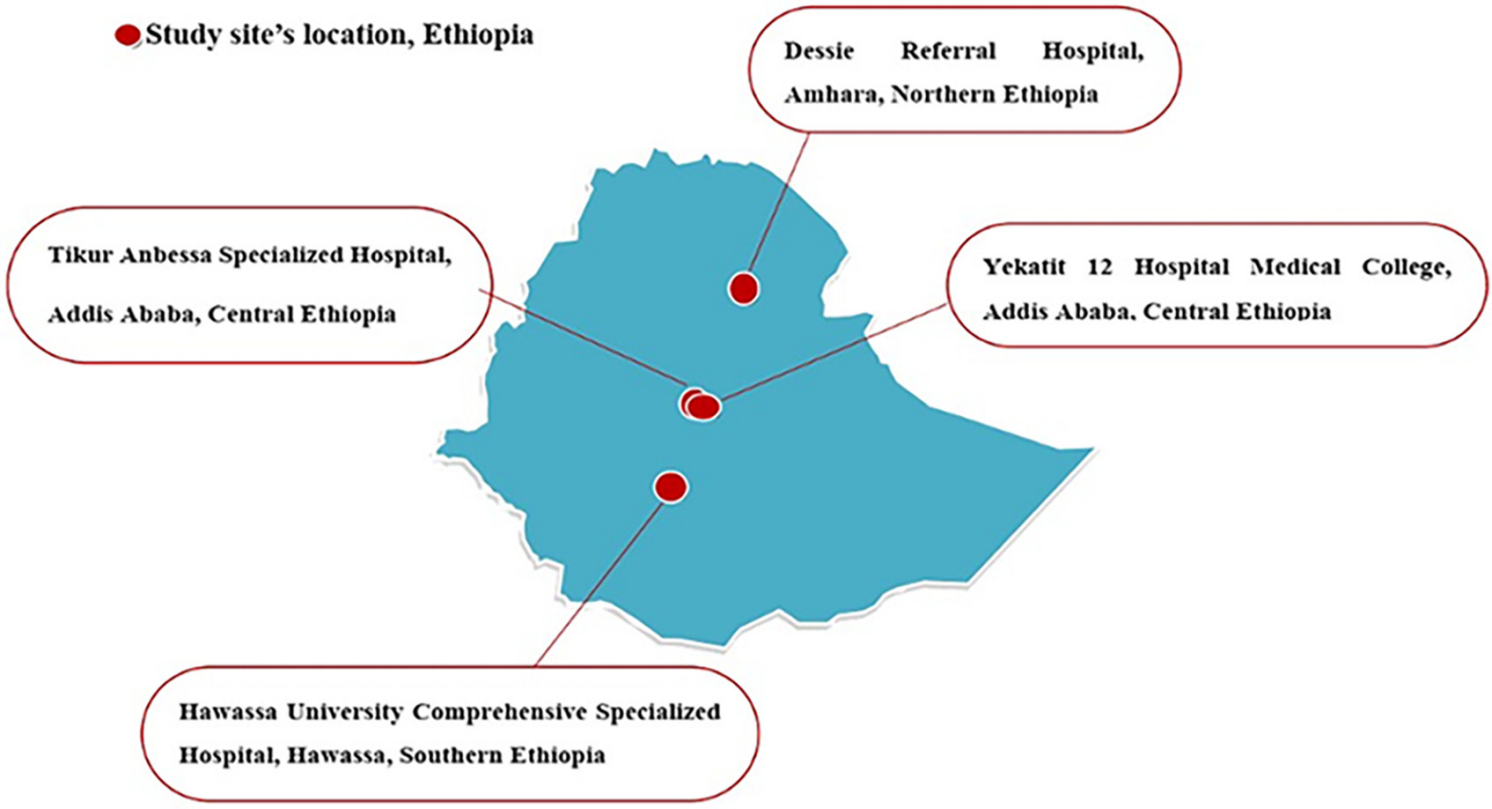
Map of the four Ethiopian referral hospitals selected for this study and where *Enterobacteriaceae* were isolated.

A multicenter prospective cross-sectional study was conducted between October 2019 and September 2020 among patients investigated for sepsis at four selected hospitals located in central, southern, and northern parts of Ethiopia (Fig. 6). These were Tikur Anbessa Specialized Hospital (TASH) and Yekatit 12 Specialized Hospital Medical College (Y12HMC) in the central, Hawassa University Comprehensive Specialized Hospital (HUCSH) in the southern, and Dessie Referral Hospital (DRH) in the northern parts of Ethiopia. The details of each study site can be accessed from a previously published paper (46).

### Patient recruitment and sample size calculation.

All patients with suspected cases of sepsis who sought medical service at the study sites were included in the study population. An attending physician’s decision was used to identify eligible patients as sepsis cases. All age groups were included, but patients who had been on antibiotic treatment within the preceding 10 days were excluded from the study. The sociodemographic, clinical, and risk factor data of eligible patients were gathered by using a standardized pretested questioner. A total of 1,416 clinically diagnosed cases of sepsis from different wards were enrolled in the study. The sample size was calculated based on a single sample size estimation formula, *n* = *Z*^2^*P*((1 − *P*)/*d*^2^), using a proportion (*P*) of 50% (*P* = 0.5), due to lack of previous similar multicenter study. As this was a prospective multicenter study, increasing the sample size was necessary; hence, a precision (*d*) of 0.03 was used to maximize the sample size. *Z* stands for Z statistic with the level of confidence of 95%, which is conventional where the *Z* value is 1.96. With a 10% nonresponse rate, the total sample size came to 1,174 and was distributed equally across the four study sites. Keeping the minimum sample size allocated to each study site, a total of 1,416 patients clinically investigated for sepsis were enrolled to determine the genomic epidemiology of carbapenemase-producing and colistin-resistant *Enterobacteriaceae*.

### Blood culture, isolation, and identification of *Enterobacteriaceae*.

From all study sites, a total of 1,416 clinically diagnosed cases of sepsis from different wards were enrolled in this study. A single blood culture bottle system was processed from all patients, and bacterial identification was performed with standardized laboratory protocols. At each study site, *Enterobacteriaceae* isolates were characterized by their colony characteristics, Gram staining, and conventional biochemical tests. All strains were stored at −70°C or −16°C and transported to the Armauer Hansen Research Institute and later brought to Sweden for further characterization. All *Enterobacteriaceae* isolates were reidentified and confirmed using matrix-assisted laser desorption ionization–time of flight mass spectrometry (MALDI-TOF MS) at the Clinical Microbiology Department of Uppsala University Hospital, Uppsala, Sweden, and Karolinska Institute, Stockholm, Sweden. A total of 301 *Enterobacteriaceae* isolates were subjected to whole-genome sequencing (WGS) for the current analysis.

### Carbapenem susceptibility testing using disk diffusion.

At each study site, the susceptibility of all *Enterobacteriaceae* against meropenem was analyzed using disk diffusion after 16 to 18 h of incubation at 37°C. All *Enterobacteriaceae* that showed reduced susceptibility for meropenem were retested for meropenem (10 μg) and further tested against imipenem (10 μg) and ertapenem (10 μg). All carbapenem disks were Oxoid products (Oxoid Ltd., UK). Each zone of inhibition was measured to the nearest millimeter and interpreted as sensitive, intermediate, or resistant based on the standardized table supplied by the Clinical and Laboratory Standards Institute (CLSI) (47). Using a sterile wire loop, 3 to 5 pure colonies were picked and emulsified in nutrient broth (Oxoid). Standard inoculums were adjusted to a 0.5 McFarland standard and swabbed onto Mueller-Hinton agar (Oxoid). An *Enterobacteriaceae* isolate that showed resistance or intermediate level of susceptibility to any of the three carbapenem drugs tested was selected as suspicious for carbapenemase production.

### Carbapenem MIC determination using broth dilution.

All *Enterobacteriaceae* isolates that showed resistance or intermediate level of susceptibility against meropenem (10 μg) were further investigated using standard broth microdilution. A 5-μg/mL concentration of meropenem stock solution was prepared in methanol and distilled water. A working solution of meropenem was prepared with a concentration of 64 μg/mL by adding 40 μL of stock solution to 3.96 mL of Mueller-Hinton broth 2 (Oxoid Ltd., UK). Using the 64-μg/mL working solution, desired working solutions with concentrations of 0.065, 0.125, 0.25, 0.5, 1, 2, 4, 8, 16, and 32 were prepared by 2-fold serial dilutions into the broth. From each working solution, 50 μL was transferred to a 96-well plate (Tarson microtest plate). A bacterial suspension adjusted to a 0.5 McFarland standard in 5 mL of Mueller-Hinton broth 2 was added to each well that contained meropenem working solution and incubated at 37°C for 16 to 20 h. The MIC was recorded as the lowest concentration of drug that completely inhibited visible growth. A cutoff value provided by CLSI was used to interpret the meropenem MIC result.

### Carbapenem susceptibility testing using Etest strip.

All *Enterobacteriaceae* isolates that were tested against carbapenems by using broth microdilution were further tested using Etest MIC strips of meropenem, imipenem, and ertapenem. A suspension of each *Enterobacteriaceae* adjusted to a 0.5 McFarland standard was swabbed onto Mueller-Hinton agar. Meropenem, imipenem, and ertapenem Etest MIC strips (bioMérieux, France) were placed over the swabbed plates and incubated at 37°C for 16 to 18 h. A cutoff value provided by the CLSI was used to interpret the MIC result as sensitive, intermediate, and resistant to meropenem, imipenem, and ertapenem.

### MIC determination for colistin susceptibility.

Since colistin is the last-resort antibiotic for the treatment of carbapenem-resistant *Enterobacteriaceae*, all *Enterobacteriaceae* isolates that showed a resistant or intermediate level of susceptibility against meropenem (10 μg) were investigated for the presence of colistin resistance using standard broth microdilution. A colistin sulfate salt stock solution with a concentration of 1 μg/μL (50 mg; Sigma-Aldrich) was prepared in distilled water. A working solution of colistin sulfate salt was prepared with a concentration of 16 μg/mL by adding 160 μL of stock solution to 9.86 mL broth. Using the 16-μg/mL working solution, desired working solutions with concentrations of 0.5, 1, 2, 4, and 8 μg/mL were prepared by 2-fold serial dilutions in Mueller-Hinton broth 2. From each colistin sulfate working solution, 50 μL was transferred to a 96-well plate (Tarson microtest plate). A bacterial suspension adjusted to a 0.5 McFarland standard in 5 mL of Mueller-Hinton broth 2 was added to each well, and the plate was incubated at 37°C for 16 to 20 h. The MIC was recorded as the lowest concentration of drug that completely inhibited visible growth. A cutoff value provided by CLSI was used to interpret the MIC result.

### DNA extraction and WGS.

All *Enterobacteriaceae* isolates (*n* = 301) were subjected to WGS regardless of their meropenem susceptibility status performed at each study site to avoid any phenotypic screening limitations. From all *Enterobacteriaceae* isolates, DNA was extracted manually using QIAamp DNA minikit (Qiagen, Germany) according to the manufacturer’s instructions. DNA extractions were done by taking 2 to 5 pure colonies that grew on cystine lactose electrolyte-deficient agar at 37°C for 24 h aerobically. After extraction, the DNA concentrations were measured with a Qubit 3.0 (Thermo Scientific, MA, USA). All extracted DNA samples were kept at −20°C until they were submitted for whole-genome sequence determination. All *Enterobacteriaceae* isolates were subjected to WGS at Science for Life Laboratory, Solna, Sweden. From each DNA sample (average of 10 ng), 20 μL was transferred into a 96-well WGS plate. Sequencing libraries were generated using Nextera XT (Illumina kits), and short-read sequencing was run on Illumina (HiSeq 2500) systems with a paired-end sequencing protocol (150-bp insert size) at Science for Life Laboratory.

### Genome assembly and data analysis.

SPAdes (version 3.9) was used for genome assembly. Using the assembled genomes, acquired carbapenemase and colistin resistance genes were identified using ResFinder 4.1 at the Center for Genomic Epidemiology (CGE) (https://cge.cbs.dtu.dk/services/ResFinder/) with a 90% threshold and 60% coverage. Similarly, the presence of a chromosomal point mutation that mediated resistance for carbapenems and colistin, sequence types, and plasmid replicons were identified using CGE tools. Salmonella species sequence typing was done using the SalmcgMLST v1.0 tool available at the Salmonella typing database (https://pubmlst.org/bigsdb?db=pubmlst_salmonella_seqdef). Single nucleotide polymorphism (SNP) variant calling, SNP filter, site validation, and inferring the phylogeny were done using CSI Phylogeny 1.4 (48; https://cge.cbs.dtu.dk/services/CSIPhylogeny/). Visualization of trees and metadata was done using iTOL v6.5.2 (31; https://itol.embl.de/). Plasmid replicon linkage with *bla*_NDM_ genes was created using Geneious v2022.0 (https://www.geneious.com).

### Ethical approval.

The study was approved by the Department of Microbiology, Immunology and Parasitology Ethical Review Committee (DEREC/18/19/01-H) and Institutional Review Board (AAUMF 01-008) of College of Health Sciences, Addis Ababa University. The study was also approved by AHRI/ALERT Ethics Review Committee (protocol number P050/18) of the Armauer Hansen Research Institute and the National Ethical Review Committee (reference number MoSHE/RD/14.1/690/19). Written informed consent was obtained from all patients involved in the study.

### Statistical analysis.

The data were prepared using Microsoft Office Excel and imported to SPSS v28 for analysis. The frequencies of reduced susceptibility to carbapenems by using disk diffusion, broth, and Etest MIC were calculated as well as the frequencies of colistin susceptibility using broth dilution. The frequencies of acquired genes encoding carbapenem and colistin resistance, as well as chromosomal point mutations for carbapenem and colistin resistance, were calculated. Binary logistic regression analysis was used to determine associations of sociodemographic and possible risk factors of carbapenemase-producing *Enterobacteriaceae*. *P* values of <0.05 were considered statistically significant.

### Data availability.

The genomic sequence data were submitted to the National Center for Biotechnology Information (BioProject accession no. PRJNA787062).
