# Supplementary material for: Genomic Epidemiology of Carbapenemase-Producing and Colistin-Resistant Enterobacteriaceae among Sepsis Patients in Ethiopia: a Whole-Genome Analysis
Source: Antimicrob Agents Chemother. 2022 Jul 25;66(8):e00534-22. doi: 10.1128/aac.00534-22 (PMC9380574; doi:10.1128/aac.00534-22)
Supplement: Supplemental file 1 — Fig. S1 and S2 and Tables S1 and S2. Download aac.00534-22-s0001.pdf, PDF file, 0.2 MB [file aac.00534-22-s0001.pdf]

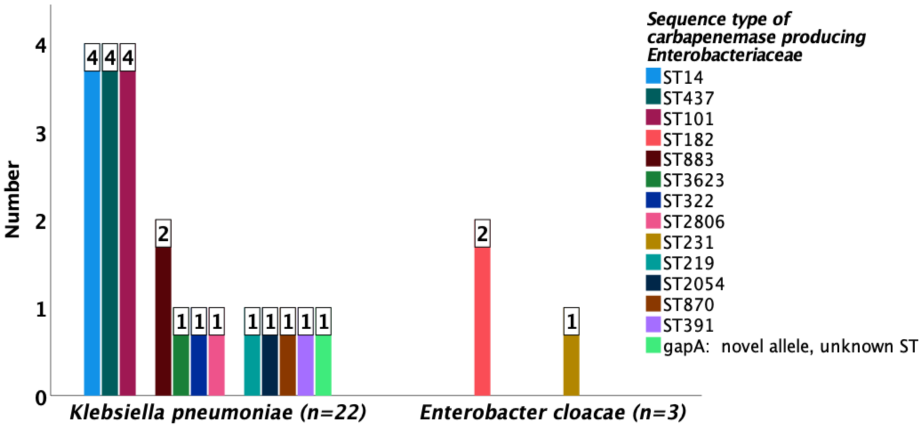

Fig. S1. Sequence type of carbapenemase producing *Enterobacteriaceae* isolated from sepsis patients in Ethiopian referral hospitals.

.

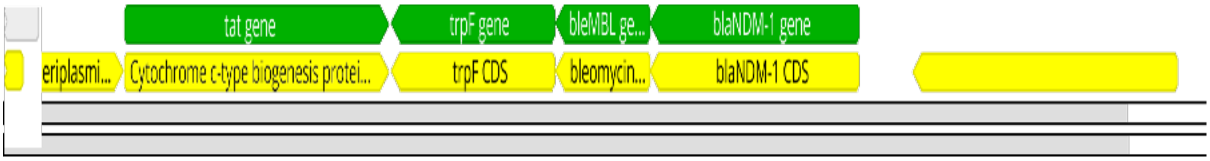

Fig. S2**.** Plasmid replicon linkage with *bla*$_{\text{NDM-1}}$ gene which was created using Geneious version 2022.

Sociodemographic data		Number	Percentage
Hospitals	TASH	501	35.4
	Y12HMC	298	21
	DRH	301	21.3
	HUCSH	316	22.3
Gender	Male	783	55.3
	Female	633	44.7
Age category	<29 days	586	41.4
	≥30 days to ≤1 year	256	18.1
	1–5 years	135	9.5
	5–18 years	158	11.2
	≥18 years	281	19.8
Ward	EOPD	104	7.3
	ICU	38	2.7
	Medical ward	148	10.5
	NICU	596	42.1
	Paediatrics	497	35.1
	Surgical ward	33	2.3
Referral patient*	Yes	722	51
	No	694	49
Previous admission	Yes	299	21.1
	No	1117	78.9
Hospital stay duration	1 week	828	58.5
	2 weeks	222	15.7
	3 weeks	146	10.3
	4 weeks and above	220	15.5
Underlying diseases	Yes	665	47
	No	751	53
Previous antibiotic treatment before recruitment to the study	Yes	440	31.1
	No	976	68.9
Fever	Up to 3 days	687	64.4
	4–6 days	207	19.4
	7 days and above	173	16.2
	No	349	24.6
	Total	1416	100

Table S1. Sociodemographic data of patients investigated for sepsis at four different hospitals in Ethiopia.

TASH – Tikur Anbessa Specialized Hospital; Y12HMC – Yekatit 12 Specialized Hospital Medical College; DRH – Dessie Referral Hospital, HUCSH – Hawassa University Comprehensive Specialized Hospital; EOPD – emergency outpatient department; ICU –

*intensive care unit; NICU – neonatal intensive care unit; * Patients who were transferred from other health care facilities to the study sites*

Hospitals	*Enterobacteriaceae*	aac(3)-IIa	aac(6')-Ib-cr	aac(6')-Ib	aac(6')-IIc	aph(3")-Ib	aph(6)-Id	ant(3")-Ia	ant(2")-Ia	aph(3')-Ia	rmtB	aadA2	aadA16	aac(3)-IId	aac(6)-Iaa
TASH	*K. pneumoniae*		Yes									Yes		Yes	
TASH	*K. pneumoniae*	Yes	Yes			Yes	Yes			Yes		Yes			
TASH	*K. pneumoniae*														
TASH	*K. pneumoniae*	Yes		Yes		Yes	Yes								
TASH	*K. pneumoniae*	Yes	Yes			Yes	Yes								
TASH	*K. pneumoniae*	Yes		Yes		Yes	Yes								
TASH	*K. pneumoniae*	Yes		Yes		Yes	Yes								
TASH	*K. pneumoniae*	Yes						Yes							
TASH	*K. pneumoniae*									Yes	Yes	Yes			
TASH	*K. pneumoniae*									Yes	Yes	Yes			
TASH	*K. pneumoniae*											Yes			
TASH	*K. pneumoniae*	Yes	Yes			Yes	Yes								
TASH	*K. pneumoniae*		Yes									Yes	Yes	Yes	
TASH	*K. pneumoniae*		Yes			Yes	Yes	Yes							
TASH	*K. pneumoniae*											Yes			
TASH	*E. cloacae*											Yes			
TASH	*E. cloacae*	Yes	Yes					Yes							
TASH	*E. cloacae*	Yes	Yes					Yes							
Y12HMC	*K. pneumoniae*	Yes	Yes			Yes	Yes		Yes						
Y12HMC	*K. pneumoniae*	Yes	Yes			Yes	Yes								
Y12HMC	*K. pneumoniae*	Yes				Yes	Yes								
Y12HMC	*Salmonella spp.*				Yes	Yes	Yes								Yes
DRH	*K. variicola*	Yes													
DRH	*K. pneumoniae*		Yes		Yes	Yes	Yes	Yes							
DRH	*K. pneumoniae*			Yes		Yes	Yes							Yes	
DRH	*K. pneumoniae*			Yes		Yes	Yes							Yes	
DRH	*K. variicola*														
HUCSH	*K. pneumoniae*	Yes	Yes					Yes		Yes			Yes		

Hospitals	*Enterobacteriaceae*	blaCTX-M-15	blaTEM-1B	blaTEM-1A	blaTEM-219	blaSHV-28	blaSHV-187	blaLEN2	blaCMY-6	blaACT-7	blaACT-16	blaSCO-1	blaLAP-2	mcr-9	catB3	cmlA1	dfrA12	dfrA14	dfrA16	dfrA19	dfrA27
TASH	*K. pneumoniae*	Yes	Yes				Yes												Yes		Yes
TASH	*K. pneumoniae*	Yes					Yes								Yes		Yes				
TASH	*K. pneumoniae*						Yes														
TASH	*K. pneumoniae*	Yes	Yes			Yes	Yes		Yes			Yes						Yes			
TASH	*K. pneumoniae*	Yes	Yes				Yes		Yes						Yes			Yes			
TASH	*K. pneumoniae*	Yes	Yes				Yes		Yes						Yes			Yes			
TASH	*K. pneumoniae*	Yes	Yes						Yes			Yes						Yes			
TASH	*K. pneumoniae*	Yes					Yes									Yes		Yes			
TASH	*K. pneumoniae*	Yes	Yes				Yes										Yes				
TASH	*K. pneumoniae*	Yes	Yes				Yes										Yes				
TASH	*K. pneumoniae*	Yes					Yes										Yes				
TASH	*K. pneumoniae*	Yes	Yes				Yes		Yes						Yes			Yes			
TASH	*K. pneumoniae*	Yes	Yes				Yes												Yes		Yes
TASH	*K. pneumoniae*	Yes		Yes	Yes		Yes								Yes			Yes			
TASH	*K. pneumoniae*	Yes					Yes										Yes				
TASH	*E. cloacae*										Yes		Yes				Yes				
TASH	*E. cloacae*	Yes								Yes	Yes	Yes			Yes	Yes					
TASH	*E. cloacae*	Yes								Yes	Yes	Yes			Yes	Yes					
Y12HMC	*K. pneumoniae*	Yes					Yes								Yes			Yes			
Y12HMC	*K. pneumoniae*	Yes	Yes				Yes						Yes		Yes			Yes			
Y12HMC	*K. pneumoniae*	Yes					Yes											Yes			
Y12HMC	*Salmonella spp.*	Yes												Yes						Yes	
DRH	*K. variicola*	Yes	Yes					Yes				Yes									
DRH	*K. pneumoniae*	Yes	Yes				Yes							Yes						Yes	
DRH	*K. pneumoniae*	Yes	Yes			Yes	Yes		Yes									Yes			
DRH	*K. pneumoniae*	Yes	Yes			Yes	Yes		Yes									Yes			
DRH	*K. variicola*							Yes													
HUCSH	*K. pneumoniae*	Yes		Yes	Yes		Yes								Yes			Yes			Yes

Hospitals	*Enterobacteriaceae*	dfrA30	mph(A)	ere(A)	QnrB1	QnrB6	qnrS1	aac(6')-Ib-cr2	tet(A)	tet(D)	sul1	sul2	ARR3	fosA	fosA6	OqxB	OqxA	qacE
TASH	*K. pneumoniae*							Yes		Yes	Yes	Yes	Yes	Yes		Yes	Yes	Yes
TASH	*K. pneumoniae*		Yes					Yes			Yes	Yes		Yes		Yes	Yes	Yes
TASH	*K. pneumoniae*														Yes	Yes	Yes	
TASH	*K. pneumoniae*				Yes			Yes	Yes		Yes	Yes			Yes	Yes	Yes	Yes
TASH	*K. pneumoniae*				Yes			Yes	Yes		Yes	Yes		Yes		Yes	Yes	Yes
TASH	*K. pneumoniae*				Yes				Yes		Yes	Yes		Yes		Yes	Yes	Yes
TASH	*K. pneumoniae*				Yes				Yes		Yes	Yes			Yes	Yes	Yes	Yes
TASH	*K. pneumoniae*			Yes	Yes						Yes		Yes			Yes	Yes	
TASH	*K. pneumoniae*	Yes	Yes								Yes				Yes	Yes	Yes	Yes
TASH	*K. pneumoniae*	Yes	Yes								Yes				Yes	Yes	Yes	Yes
TASH	*K. pneumoniae*	Yes									Yes				Yes	Yes	Yes	Yes
TASH	*K. pneumoniae*				Yes			Yes	Yes		Yes	Yes			Yes	Yes	Yes	Yes
TASH	*K. pneumoniae*					Yes				Yes	Yes	Yes	Yes		Yes	Yes	Yes	Yes
TASH	*K. pneumoniae*							Yes				Yes						
TASH	*K. pneumoniae*	Yes									Yes				Yes	Yes	Yes	Yes
TASH	*E. cloacae*		Yes				Yes				Yes							Yes
TASH	*E. cloacae*			Yes	Yes				Yes		Yes		Yes					Yes
TASH	*E. cloacae*			Yes	Yes		Yes	Yes	Yes		Yes		Yes					Yes
Y12HMC	*K. pneumoniae*						Yes	Yes				Yes						
Y12HMC	*K. pneumoniae*		Yes		Yes			Yes	Yes			Yes			Yes	Yes	Yes	
Y12HMC	*K. pneumoniae*						Yes											
Y12HMC	*Salmonella spp.*			Yes						Yes	Yes	Yes						Yes
DRH	*K. variicola*																	
DRH	*K. pneumoniae*			Yes				Yes			Yes	Yes		Yes		Yes	Yes	Yes
DRH	*K. pneumoniae*									Yes	Yes	Yes			Yes	Yes	Yes	Yes
DRH	*K. pneumoniae*									Yes	Yes	Yes			Yes	Yes	Yes	Yes
DRH	*K. variicola*																	
HUCSH	*K. pneumoniae*		Yes				Yes	Yes			Yes		Yes					Yes

Table S2. Antimicrobial resistance genes detected among carbapenem resistant *Enterobacteriaceae* isolated from sepsis patients in four Ethiopian referral hospital.

TASH – Tikur Anbessa Specialized Hospital; Y12HMC – Yekatit 12 Specialized Hospital Medical College; DRH – Dessie Referral Hospital, HUCSH – Hawassa University Comprehensive Specialized Hospital.
